# Barriers and Enablers of Kangaroo Mother Care Practice: A Systematic Review

**DOI:** 10.1371/journal.pone.0125643

**Published:** 2015-05-20

**Authors:** Gabriel Seidman, Shalini Unnikrishnan, Emma Kenny, Scott Myslinski, Sarah Cairns-Smith, Brian Mulligan, Cyril Engmann

**Affiliations:** 1 Harvard T. H. Chan School of Public Health, Boston, Massachusetts, United States of America; 2 Boston Consulting Group, Boston, Massachusetts, United States of America; 3 Boston Consulting Group, New York City, New York, United States of America; 4 Bill & Melinda Gates Foundation, Seattle, Washington, United States of America; The Hospital for Sick Children, PAKISTAN

## Abstract

Kangaroo mother care (KMC) is an evidence-based approach to reducing mortality and morbidity in preterm infants. Although KMC is a key intervention package in newborn health initiatives, there is limited systematic information available on the barriers to KMC practice that mothers and other stakeholders face while practicing KMC. This systematic review sought to identify the most frequently reported barriers to KMC practice for mothers, fathers, and health practitioners, as well as the most frequently reported enablers to practice for mothers. We searched nine electronic databases and relevant reference lists for publications reporting barriers or enablers to KMC practice. We identified 1,264 unique publications, of which 103 were included based on pre-specified criteria. Publications were scanned for all barriers / enablers. Each publication was also categorized based on its approach to identification of barriers / enablers, and more weight was assigned to publications which had systematically sought to understand factors influencing KMC practice. Four of the top five ranked barriers to KMC practice for mothers were resource-related: “Issues with the facility environment / resources,” “negative impressions of staff attitudes or interactions with staff,” “lack of help with KMC practice or other obligations,” and “low awareness of KMC / infant health.” Considering only publications from low- and middle-income countries, “pain / fatigue” was ranked higher than when considering all publications. Top enablers to practice were included “mother-infant attachment” and “support from family, friends, and other mentors.” Our findings suggest that mother can understand and enjoy KMC, and it has benefits for mothers, infants, and families. However, continuous KMC may be physically and emotionally difficult, and often requires support from family members, health practitioners, or other mothers. These findings can serve as a starting point for researchers and program implementers looking to improve KMC programs.

## Introduction

Preterm birth is a major global health issue, with 15 million preterm births occurring each year, and over 1 million of these preterm infants dying each year [1]. Preterm birth complications directly account for greater than 35% of all neonatal deaths each year, and preterm birth indirectly contributes to an even greater percentage because it increases the risk that an infant will die from infection. Preterm births are on the rise globally, both in high-income and low-income settings [1]. The 10 countries with highest rates of preterm births include those that are high-income, such as the USA, middle-income such as India, China, the Philippines, Indonesia and Brazil, and low-income such as Nigeria, Pakistan, Bangladesh, Democratic Republic of Congo [1]. Thus interventions that are feasible and applicable in both high- and low-income settings are highly desired.

Kangaroo mother care (KMC) is an evidence-based approach to reducing mortality and morbidity in preterm infants which was first developed in Bogotá Colombia. According to the World Health Organization's definition, KMC consists of prolonged skin-to-skin (STS) contact between mother and infant, exclusive breastfeeding whenever possible, early discharge with adequate follow-up and support, and initiation of the practice in the facility and continuation at home [2]. In a meta-analysis, KMC was shown to significantly reduce preterm mortality at 40–41 weeks' corrected gestational age by 40% and to improve other outcomes including severe infection / sepsis, emotional attachment in mothers, and weight gain versus conventional neonatal care in preterm infants [3]. Another meta-analysis showed a similar mortality benefit, although it included fewer studies in its analysis [4]. Research from various countries also suggests that KMC is a cost-effective method for treating preterm infants [5,6], that mothers who have practiced KMC may find it acceptable [6–8], and that KMC can have a positive impact on the health of mothers in certain cases [9,10]. Therefore, KMC is a highly relevant intervention that should be considered for scaling across geographies. Although the WHO definition of KMC specifies that the practice should be initiated in a facility setting, several studies and trials have explored whether KMC can be effective in a community-initiated setting, and the effectiveness of KMC in this context has not yet been conclusively determined [11,12].

In spite of these benefits, mothers may face barriers to practice, some of which may prevent them from achieving the continuous STS contact with their infants (a defining feature of KMC). For example, a survey of 46 mothers of preterm infants who were trained on KMC in a facility in Andhra Pradesh, India found that only 6.5% of mothers felt that providing KMC for 12 hours / day or greater was feasible, whereas 52% of mothers felt that only 1 hour / day was practical[8]. Similarly, in a trial of community-initiated KMC with 1,565 mother-infant pairs, only 23.8% practiced STS for more than 7 hours / day in the first 48 hours of life, and the average number of hours of STS during days 3–7 of life was 2.7 ± 3.4 hours [11]. Barriers to the other components of KMC, including breastfeeding [12,13], and adequate follow-up after discharge [14,15], have also been noted.

KMC has emerged as a key intervention package for a number of newborn health initiatives, and this is epitomized by the Every Newborn Action Plan (ENAP) [16]. Additionally a recent convening of ideas from 600 key programmers, policymakers, researchers and stakeholders in newborn health, using the Child Health and Nutrition Research Initiative [CHNRI] method, highlighted KMC as a top preterm intervention agenda [17]. Many agencies, such as Save the Children's Saving Newborn Lives III (SNL), USAID, WHO and the Bill & Melinda Gates Foundation, and some countries, such as Malawi and South Africa, have also made KMC a priority [18–22].

Therefore, to adequately implement and effectively scale-up this intervention, it is critical to understand the key factors that contribute to a mother's (in)ability to practice KMC. However, there is a dearth of synthesized information on all of the sociocultural, resourcing, and experiential factors that influence a mother's practice of KMC. Accordingly, this review sets out to synthesize existing literature on the factors which influence a mother's ability to practice KMC by answering two questions. First, what are the most frequently cited barriers that could prevent a mother from successfully practicing KMC? These barriers can exist at multiple levels, including barriers to implementation of a KMC program, deficiencies in the program itself, or specific challenges associated with the practice of KMC which the mother has to perform. Second, are there any key positive factors, cited in the relevant literature, that can enable a mother to practice KMC? We believe that it is of utmost importance to consider these different types of barriers together (along with key enablers to practice), even though the solutions for solving each barrier might be different. Even though the specific barriers most relevant for mothers may vary based on context, a comprehensive list of this type will give program implementers, policymakers, and researchers a synthesized set of factors to consider as they attempt to implement new or improve existing KMC programs.

## Methodology

### Search strategy and selection criteria

We undertook a systematic review according to PRISMA 2009 guidelines to answer these two questions [23]. (See S1 Appendix for complete PRISMA checklist). We developed a review protocol with methods and eligibility criteria that were specified in advance. We included any publication in our study that met the following criteria: 1) the aim of the study was to document experiences implementing KMC, STS, or other interventions related to Reproductive, Maternal, Newborn, & Child Health and Nutrition (RMNCH&N) that may have included KMC / STS, or the publication had relevant information on specific barriers to implementation listed in the abstract; 2) the study was published in a peer-reviewed journal; 3) the study included data on the sample population, sample size, and location of implementation; 4) the study was original research; and 5) the study was published in English. Studies testing the efficacy of KMC or STS practice (e.g. randomized controlled trials) were included if issues of acceptability, feasibility, or barriers to practice for parents or practitioners were documented in the abstract. Any publication published before August 13, 2013 (the date of the final database search) was eligible for inclusion. We excluded literature reviews, conference proceedings, letters to the editor, and abstracts in order to prevent double counting of data and to ensure that all barriers were understood in the context of the entire study.

We searched nine electronic databases: PubMed, EMBASE, Scopus, Web of Science, and the WHO Regional Databases (AIM, LILACS, IMEMR, IMSEAR, and WPRIM). We searched all databases using the following search terms: "Kangaroo Mother Care" OR "Kangaroo Care" OR "Skin to skin care". In addition, because at least one relevant article identified from a list of references in a literature review included the terms Kangaroo Mother Care in quotations and the term Skin to skin, we also searched PubMed for "'Kangaroo Mother Care'" and "Skin to skin". We used broad search criteria to ensure that relevant articles were not missed, and we then filtered and excluded many articles based on the eligibility criteria mentioned above. Reference lists from literature reviews identified in the database search were also scanned for relevant titles, and articles were also identified in consultation with the authors on this study. Recommendations for studies to be included in the review were also received from participants at the KMC Acceleration Meeting in Istanbul, October 2013[24] and in consultation with leaders in the fields of KMC and newborn health.

### Data collection

After our initial database search and identification of additional studies through recommendations and scans of reference lists, study titles and abstracts were screened by two reviewers (GS and EK) for inclusion. In situations when a study's eligibility was disputed, a third reviewer (SU) provided an independent assessment until consensus was reached.

96 articles were reviewed to identify a comprehensive list of barriers to KMC practice in advance of the KMC Acceleration Convening [24]. A data extraction sheet was piloted and tested using these 96 articles. This piloting allowed for preliminary identification of relevant barriers and enablers to be included in the final review as well as final determination of stakeholders to be included in the review: mothers, fathers, community health workers, nurses, physicians, and program managers. The final tool included fields for collecting publication details, relevant study characteristics (sample size, location, and a short description of each study), barriers for each stakeholder group, and enablers to practice for mothers. Results from the preliminary analysis were shared at the KMC Acceleration Convening, ensuring that key stakeholders in the KMC community generally supported the methodology (described in further detail in the next section) and found the preliminary results to be consistent with their experiences [24]. This convening included researchers and practitioners from many different low- and middle-income countries (LMIC) across Latin America, Sub-Saharan Africa, and Asia, as well as major foundations and civil society organizations involved in RMNCH&N

Once the tool and list of studies was finalized, data was captured from each article into the tool independently by two reviewers (GS and EK) and a third reviewer (SU) provided independent assessment in case of disputes. The main outcome of interest was the frequency with which a barrier / enabler was mentioned across publications. Using frequency of mention allowed for a synthesized view of the barriers / enablers to practice listed in the relevant literature. The data collection process involved identifying barriers and enablers of KMC practice listed in each study (either through qualitative or quantitative findings) and categorizing them into one of the pre-determined categories of barriers / enablers in the tool. There was no limit to the number of barriers / enablers that could be found in a single study, but each study could only count toward a given barrier / enabler once. For example, if a study mentioned several statistics all indicating that mothers' low awareness of KMC was a barrier to practice, this would be coded as a single instance of low awareness among mothers in the tool. In cases where a barrier or enabler was listed for parents in general and did not distinguish between mothers and fathers, this barrier was listed as a barrier for mothers. In cases where a barrier was listed for both nurses and physicians but did not distinguish between the two, this barrier was listed as a barrier for nurses. Barriers / enablers were grouped into three different categories—resourcing, experiential, and sociocultural—based on consensus among all authors. Definitions for these three categories are included in S2 Appendix.

### Risk of bias and publication weighting methodology

The goal of this study was to synthesize existing literature on barriers to and enablers of KMC practice. As noted, there is limited systematically organized information on this topic. Therefore, in order to ensure that our review captured as many relevant qualitative and quantitative findings as possible, we chose to include any study identified through our search strategy which had information on barriers and enablers to KMC practice, even if studying this topic was not the primary purpose of the publication.

As one might expect based on this search strategy, our findings included many studies which had observational information on barriers to / enablers of KMC practice. Given the limited amount of synthesized information on barriers to KMC practice, we felt it was important to include these observational findings so that relevant programmatic experience informed this review. At the same time, however, we also sought to ensure that our analysis was weighted toward data from publications which had explicitly studied barriers to KMC practice (rather than giving those data equal weighting to observational findings).

Therefore, we developed a methodology to weight findings from each publication based on the way in which the data was identified and captured. Other public health literature reviews have used similar methods to quantify qualitative data drawn from multiple sources of varying quality and relevance [25–28]. We categorized each publication into one of four types: Indirect study, Exploratory study, Systematic study, and Prioritized study. Indirect studies were defined as those which did not set out to study barriers to / enablers of KMC practice, but which identified and documented these factors (ie, through observational findings). Exploratory studies were defined as those which set out to identify barriers / enablers to KMC practice but which did not pre-specify factors under consideration (ie, were not explicitly testing hypotheses about which barriers / enablers would influence practice). Systematic studies were defined as those which set out to identify barriers / enablers of KMC practice and which did pre-specify the factors under consideration but which did not prioritize among these barriers. Prioritized studies were defined in the same way as systematic studies with the exception that these studies also prioritized the barriers to KMC practice. Our indexed ranking methodology gave the most weight to Prioritized studies, the second-most weight to Systematic studies, the third-most weight to Exploratory studies, and the least weight to Indirect studies. (S2 Appendix provides more detail on full methodology describing indexed ranking process.) Note that in our findings and discussion, we refer to "top-ranked" barriers to practice for mothers and other groups. Top-ranked barriers are those that received the highest score based on this indexed ranking, which accounts for both frequency of mention across publications and weighting of each piece of evidence based on the publication type.

Each study was placed into one of these categories independently by two reviewers (GS and EK), and in cases of a discrepancy, a third reviewer provided an independent assessment (SU). Of the 103 publications included in this review, there were only 12 discrepancies (11.65%) in categorization between the first two readers, suggesting that this method is reliable for categorizing publications. Our data capture tool included a field to categorize each publication into one of these four categories.

## Results

### Study selection

**Fig 1:**
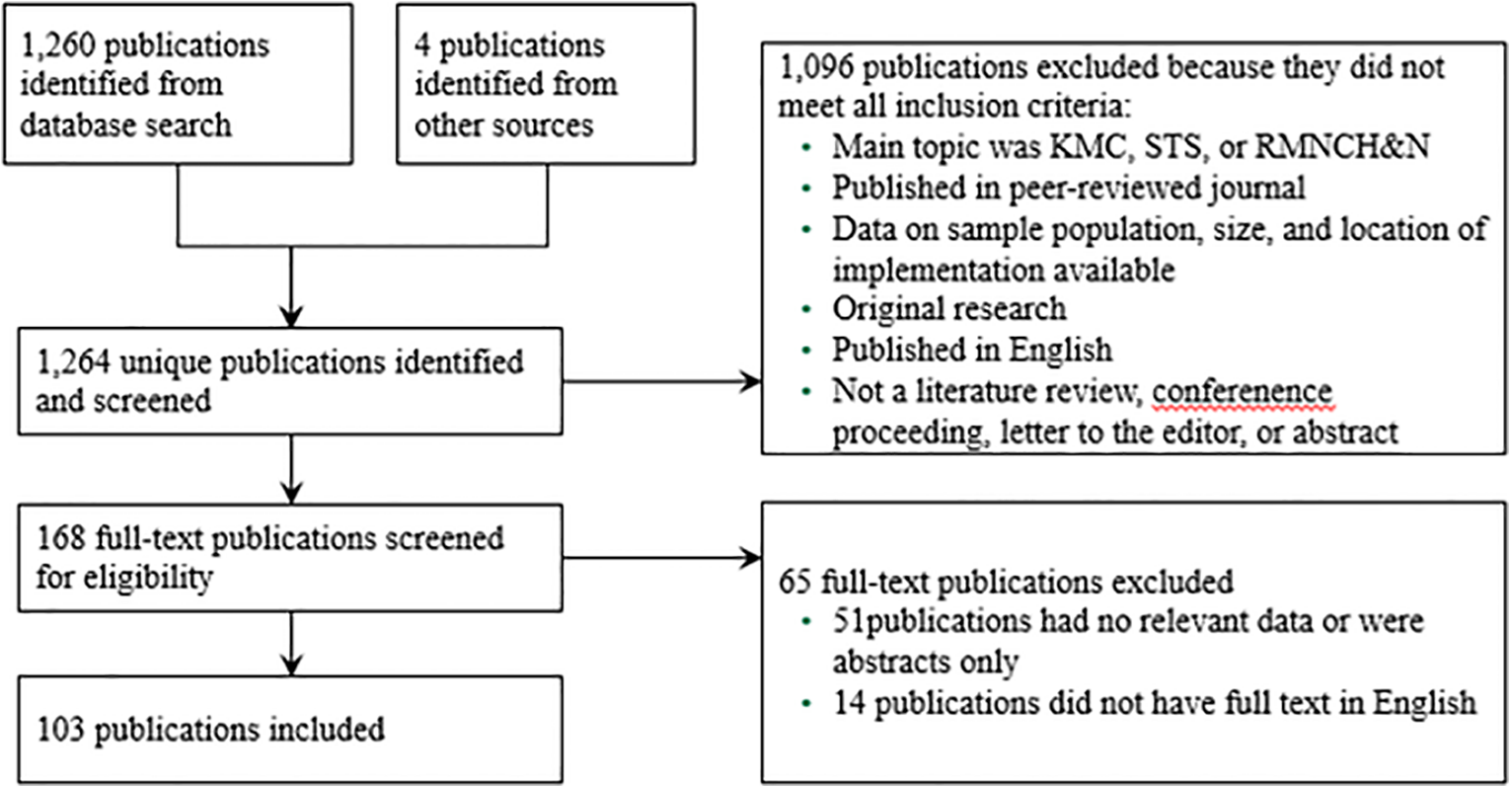
Study selection for inclusion in systematic review.

From our database search, a total of 1,260 unique publications were identified, and four others were identified through snowballing. Of these 1,264, 168 met preliminary eligibility criteria based on a scan of the title and abstract; all others were excluded because they did not meet at all eligibility criteria discussed in the Methodology section. Of these 168, 51 were eliminated after full-text screening because they did not have relevant data (i.e. barriers to newborn health intervention rollout were listed, but no barriers specific to KMC / STS were listed) or because only an abstract was available, and 14 did not have full text available in English. This resulted in 103 articles deemed relevant for inclusion in the review. Fig 1 represents the study selection for inclusion in the systematic review. A full list of publications included in this review can be found in S3 Appendix.

Of these 103 articles, 49 were from high-income countries HIC [29], 22 were from Sub-Saharan Africa, 15 were from South Asia, five were from North Africa / the Middle East, five were from Latin America / Caribbean, three were from Eastern Europe, two were from East Asia / Southeast Asia / Pacific, and two were from LMIC in multiple regions.

Nine of the publications were classified as Prioritized, 48 were classified as Systematic, 31 were classified as Exploratory, and 15 were classified as Indirect. Indirect studies included randomized controlled trials that discussed barriers to implementation and practice, two case studies of individuals' experiences with KMC, and studies on practices throughout the NICU which included information on KMC or STS practice.

A complete dataset used for analyses can be found in S1 Dataset.

### Barriers and enablers of KMC practice for mothers

**Fig 2:**
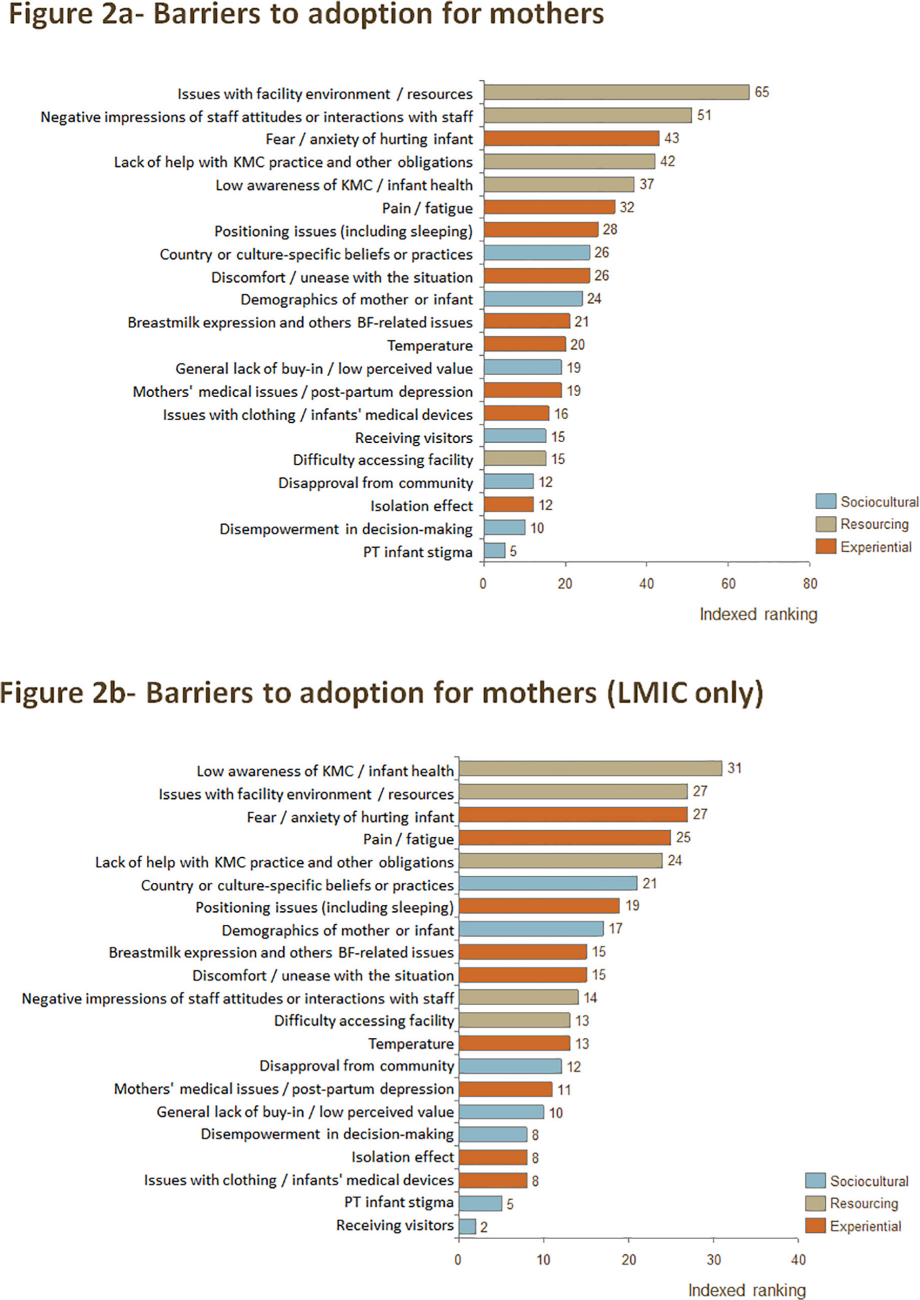
a) Indexed ranking of barriers to adoption of KMC for mothers in all countries, and b) indexed ranking of barriers to adoption of KMC for mothers in LMIC only.

Of the top five barriers to KMC practice identified for mothers, four were resource-related. The top two barriers to practice identified—"Issues with facility environment / resources" and "Negative impressions of staff attitudes or interactions"—were specific to the facility setting. "Fear / anxiety of hurting the infant," an experiential barrier to practice, was ranked third. Resource-related barriers that are relevant both inside and outside the facility—"Lack of help with KMC practice and other obligations" and "Low awareness of KMC / infant health"—were ranked fourth and fifth. When considering publications from LMIC only, four of the five top barriers were the same as when all publications were considered. The only difference is that "Negative impressions of staff attitudes or interactions" dropped significantly (to 11th), and "Pain / fatigue" emerged as the fourth-highest-ranked barrier, just after "Fear / anxiety of hurting the infant." The full rankings of barriers identified for mothers can be found in Fig 2A, and the full ranking of barriers identified for mothers from LMIC only can be found in Fig 2B.

**Fig 3:**
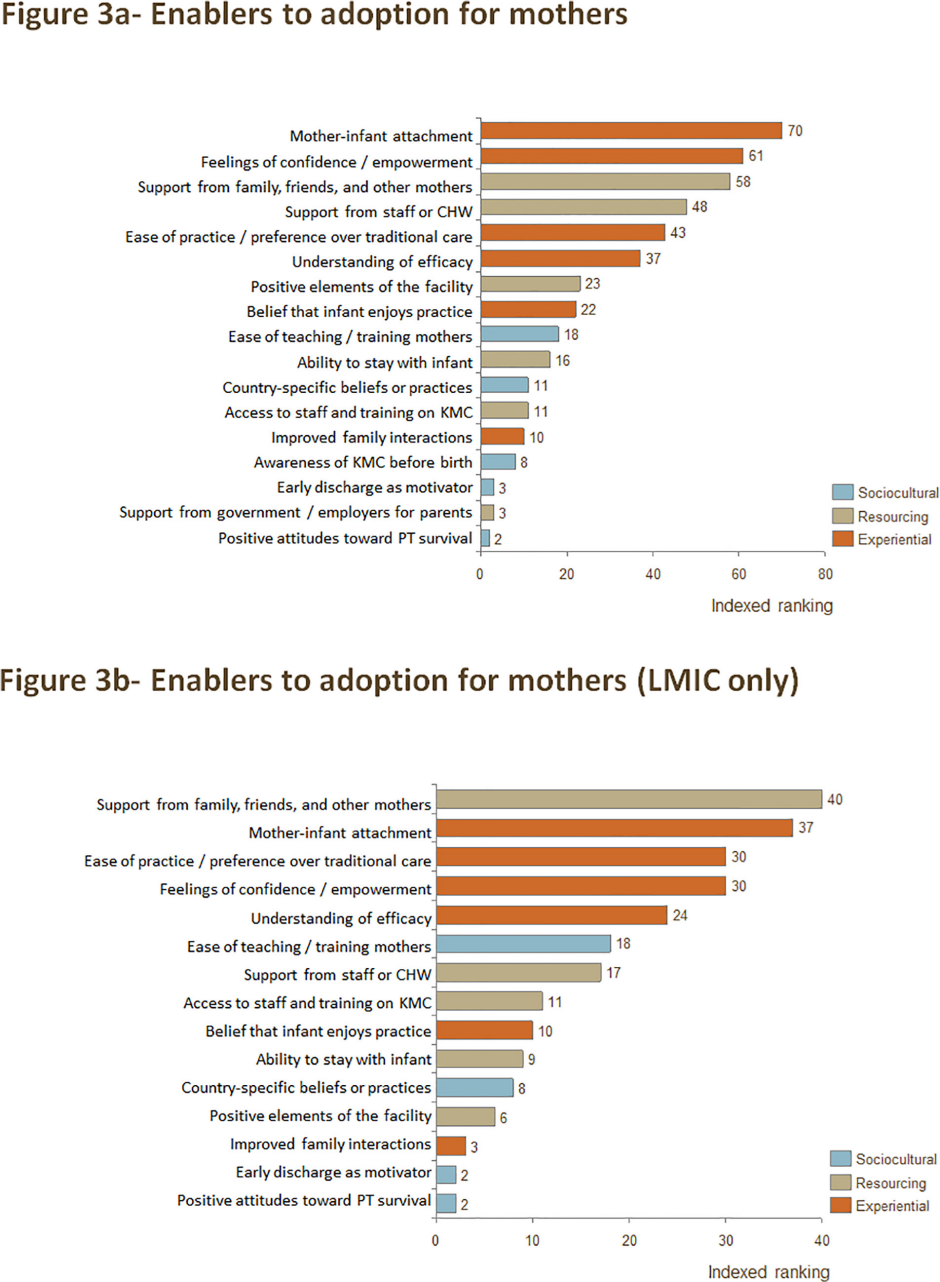
a) Indexed ranking of enablers to adoption of KMC for mothers in all countries, and b) indexed ranking of enablers to adoption of KMC for mothers in LMIC only.

Experiential factors emerged as the top enablers to KMC practice for mothers. "Mother-infant attachment," "Feelings of confidence / empowerment," and "Ease of practice / preference over traditional care" emerged as three of the top five enablers both when considering all publications and just those from LMIC. "Support from family, friends, and other mothers," a resourcing enabler, was also in the top five enablers when considering all publications, and it was the top-ranked enabler when considering publications only from LMIC. "Support from staff or community health worker (CHW)" was the fourth-highest-ranked enabler when considering all publications, but was ranked seventh when considering LMIC only. "Understanding of efficacy" was also ranked among the top five enablers to practice when considering LMIC only. The full ranking of enablers for mothers across all publications and in LMIC only can be found in Fig 3A and Fig 3B, respectively.

### Barriers of KMC for nurses

**Fig 4:**
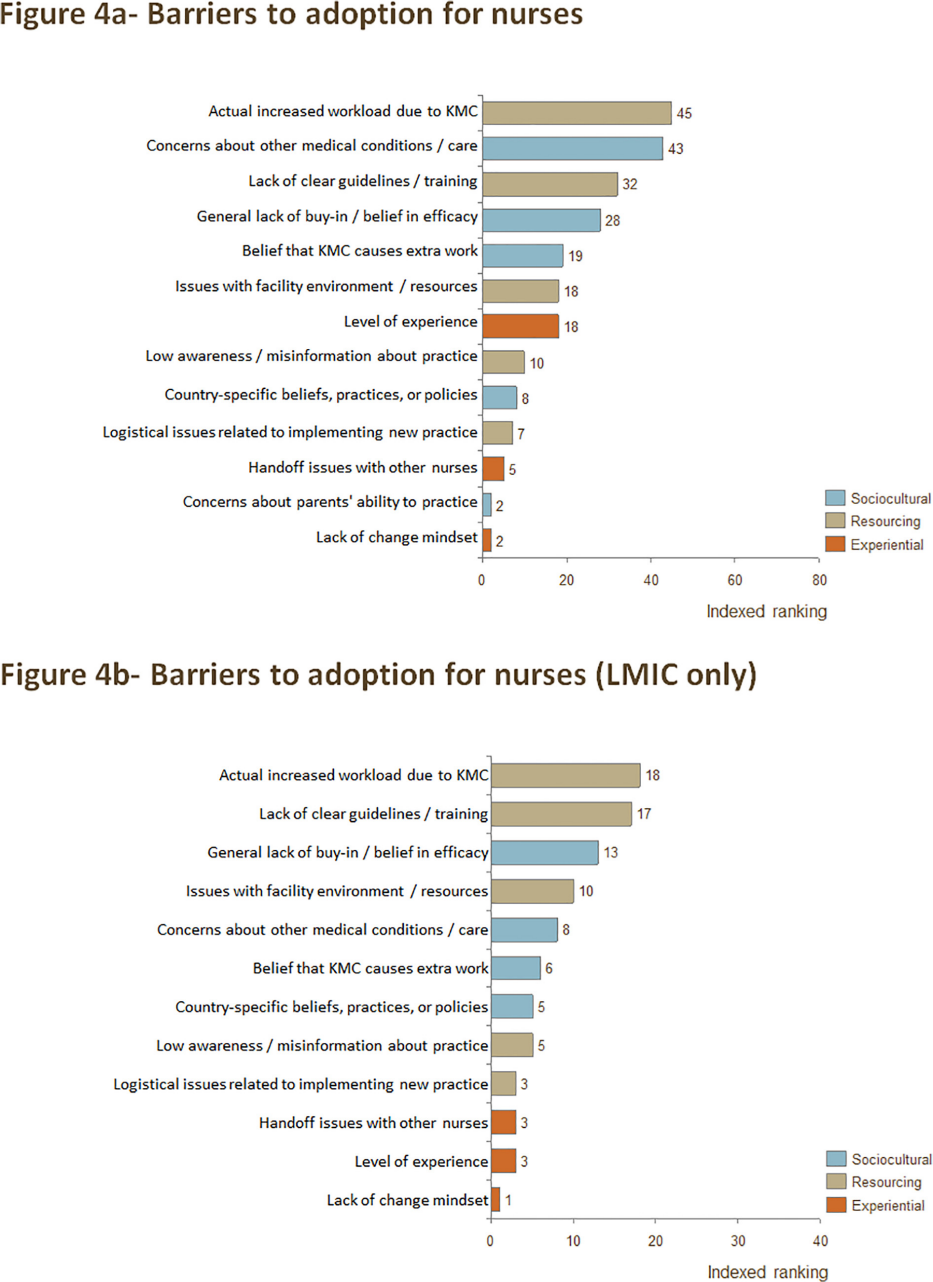
a) Indexed ranking of barriers to adoption of KMC for nurses in all countries, and b) indexed ranking of barriers to adoption of KMC for nurses in LMIC.

Resourcing and sociocultural factors emerged as the top barriers to KMC adoption for nurses. The resourcing barriers "Actual increased workload / staff shortages" and "Lack of clear guidelines / training" were in the top five barriers for nurses when considering publications from all geographies and just those from LMIC. The sociocultural barriers "General lack of buy-in / belief in efficacy" and "Concerns about other medical conditions / care" were also in the top five barriers for nurses when considering publications from all geographies and just those from LMIC. (Note that a data point was counted in the "Concerns about other medical conditions / care" category when the publication indicated that nurses' beliefs countered guidelines for KMC practice or when there was lack of consensus among nurses about whether KMC was safe to practice when an infant had a certain condition). The full ranking of barriers to adoption for nurses across all publications and in LMIC only can be found in Fig 4A and Fig 4B, respectively.

### Barriers for fathers, CHW's, physicians, and program managers

**Fig 5:**
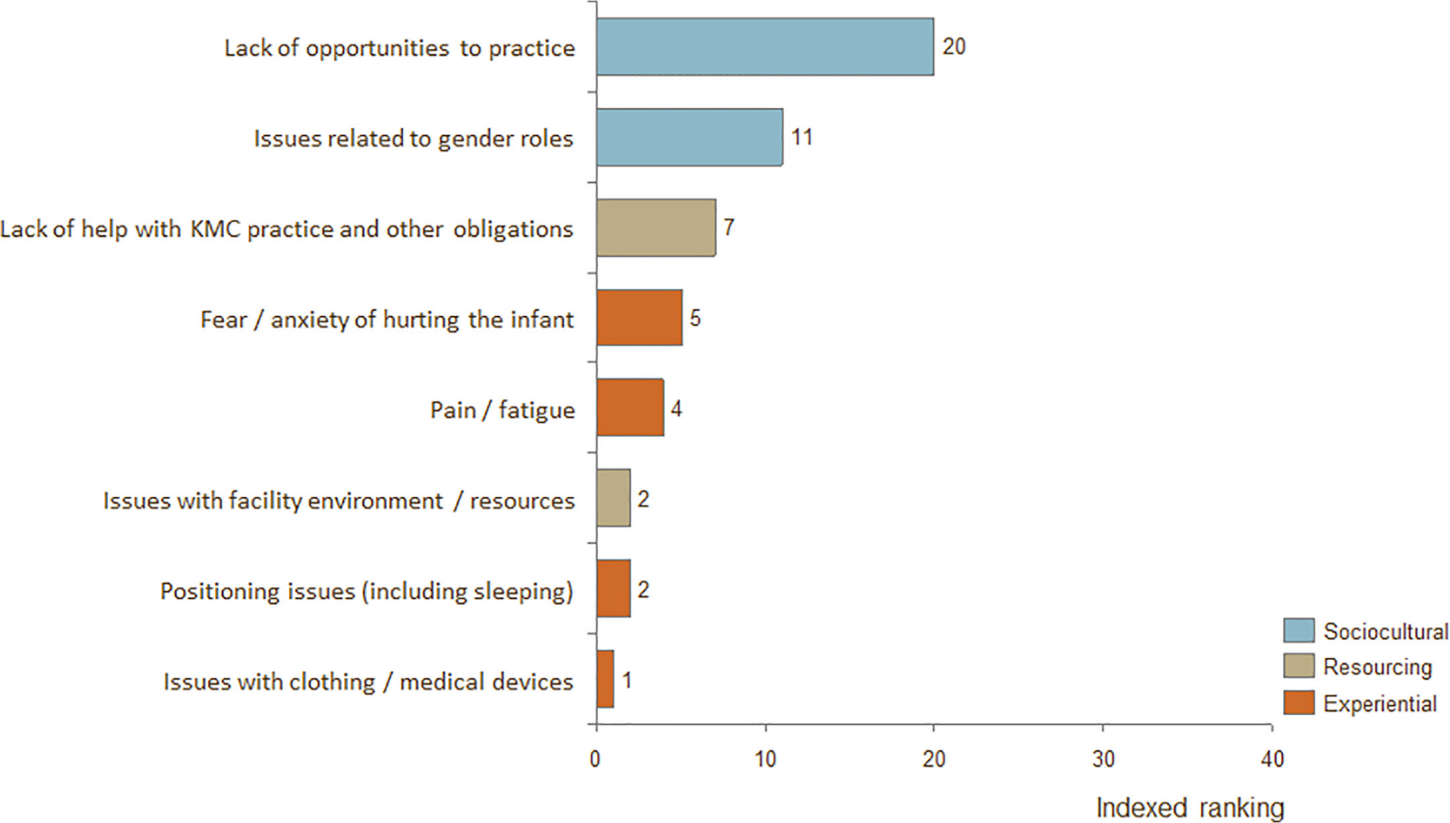
Indexed ranking of barriers to adoption of KMC for fathers in all countries.

**Fig 6:**
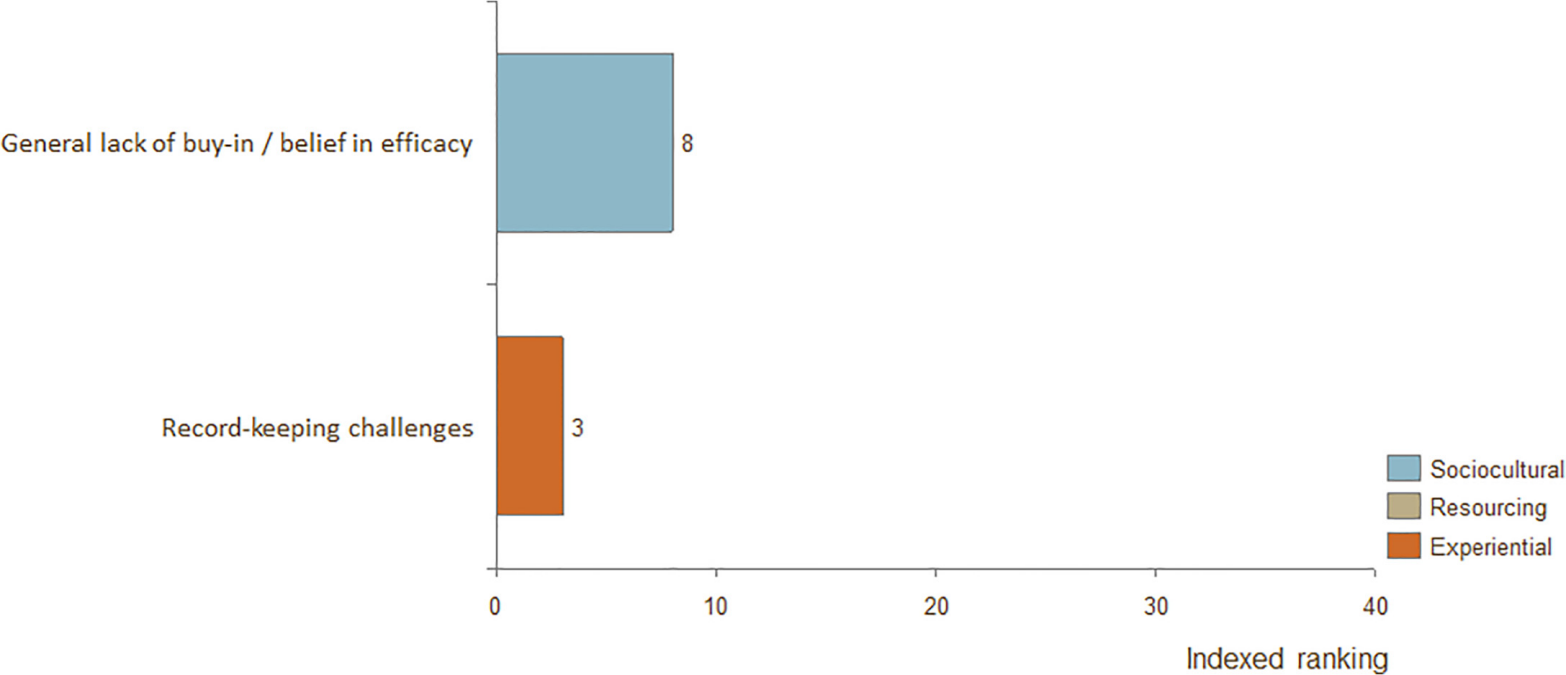
Indexed ranking of barriers to adoption of KMC for physicians in all countries.

**Fig 7:**
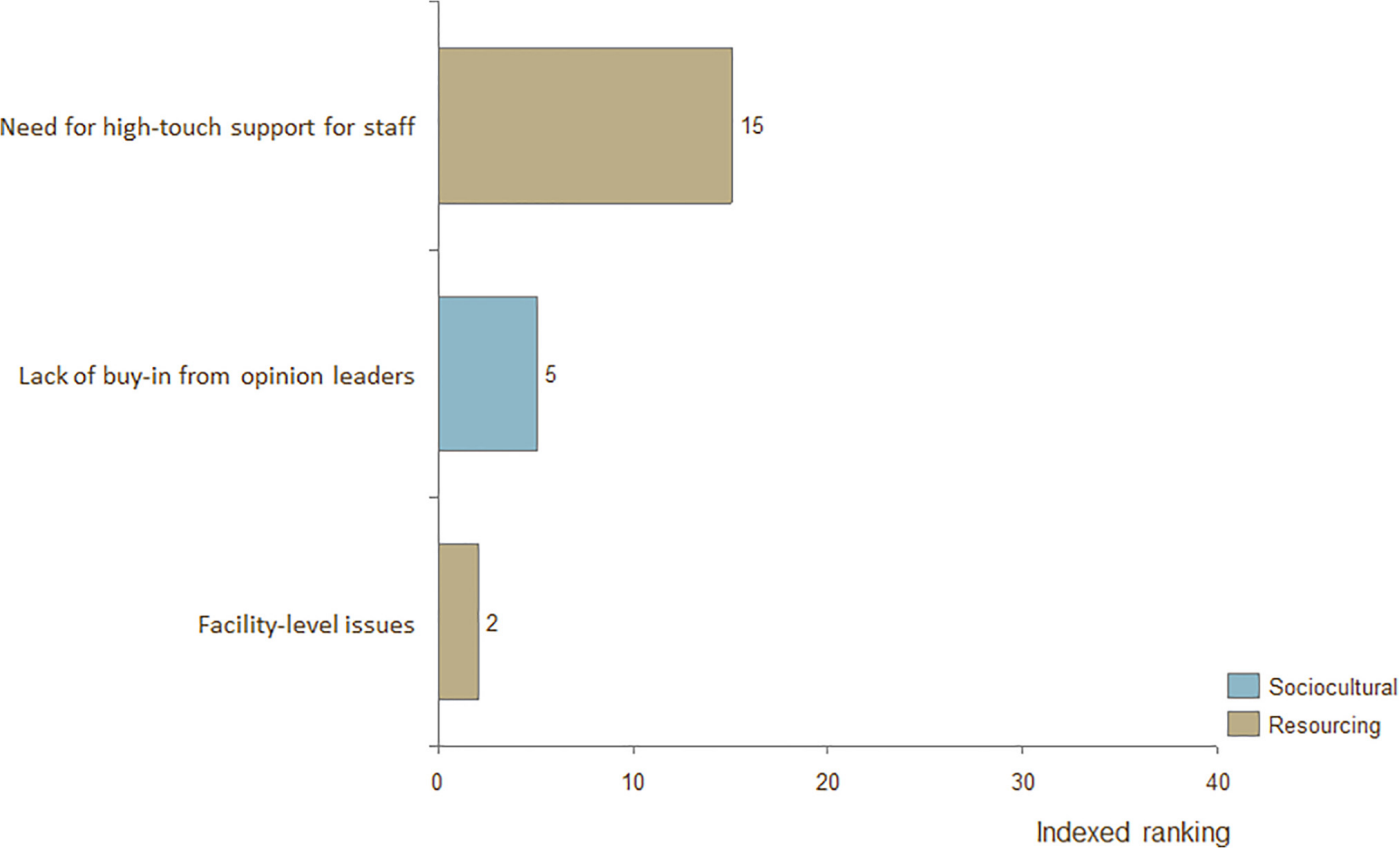
Indexed ranking of barriers to adoption of KMC for program managers in all countries.

Much less data was available for fathers, physicians, and program managers than was for mothers and nurses. Full rankings of barriers for these stakeholders across all publications can be found in Figs 5–7.

The top-ranked barrier for fathers was "Lack of opportunity to practice." The top-ranked barrier for physicians was "General lack of buy-in / belief in efficacy." The top-ranked barrier for program managers was "Need for high-touch support from staff."

## Discussion

The aim of this systematic review was to identify the most frequently cited barriers to KMC adoption, as well as enablers to practice. Given the increasing importance of KMC in addressing the global health challenge of preterm birth and death, synthesizing the experiential, resourcing, and sociocultural barriers that could prevent a mother from effectively practicing KMC is critical to effectively implementing this intervention. Although much has been written on this topic, nearly half (44.6%) of the publications identified for inclusion in this review were categorized as either Exploratory or Indirect, suggesting that there is lots of data relevant to the promotion of KMC that is not organized in a systematic way which can readily guide program implementation.

Based on the list of barriers and enablers found in the publications identified, we have identified five key insights which we believe are relevant for program implementers and researchers. Each of these insights is detailed below.

### Mothers are generally able to understand and accept KMC

Low awareness of KMC and infant health more broadly was the fourth-highest-ranked barrier to KMC practice across all publications, and the highest barrier to KMC practice when considering only publications from LMIC. However, this barrier may be over-represented in the literature on KMC because it is easily testable and many publications that implemented KMC in a new setting surveyed pre-existing levels of awareness to establish a baseline. Lack of information about KMC, hypothermia, or newborn health was identified across HIC (Sweden [30,31], Unite[32]d States[33]) and LMIC (Bangladesh [11], Egypt [34,35], Ghana [36,37], India [8,32,38], South Africa [22,39], and Zimbabwe [40]).

In spite of low general awareness of KMC, however, the literature from LMIC suggests that it is easy to train mothers on KMC practices and that they can understand the practice. For example, a training program in India found that 88% of mothers were able to understand KMC with a single training session [10]. Similarly, during site visits to facilities practicing KMC in Ghana, researchers found that mothers practicing KMC were able name its benefits [41]. Mothers were also able to understand the KMC messages delivered by community health workers in a community setting in Bangladesh [12].

Mothers' understanding of the practice also seems to enhance their adherence to practice. In South Africa, for example, mothers' "main motivation for embracing [KMC] was the well-being of their infants" [22]. Similarly, studies in Ghana found, "all mothers recognised that their babies' small weights put them at risk of illness and death and appreciated that [STS] could improve their health and survival,"[37] and, "as a motivational factor, mothers and health workers also mentioned various success stories of infants who had survived having been nursed in KMC." [41] Belief in the efficacy of KMC as an enabling factor for practice was also mentioned in HIC. One case study from the United States describes how the mother used research articles demonstrating KMC's benefits to convince facility staff to let her practice KMC [42].

### Mothers can enjoy practicing KMC, and the practice has benefits for mothers and families

Mothers not only are able to understand and accept KMC, but also they may enjoy the practice. Mother-infant attachment was the top-ranked enabler for KMC practice, and evidence for this enabler came from across HIC and LMIC. In Colombia, for example, sensitivity to infants was significantly higher among mothers practicing KMC compared to control (p<.05), and cognitive fostering was significantly higher among KMC mothers compared to control after 14 days (p<.05) [43]. Similarly, in India, KMC mothers were more likely to spend time with their baby "beyond the usual care taking" (p<.05), derive pleasure from their baby (p<.05), and only go out for "totally unavoidable" reasons (p<.05) compared to controls [44]. Qualitative findings from HIC also support these findings [13,30,45].

Several studies have shown that KMC has positive impact on the mother. Although postpartum depression can be a barrier to practicing KMC [46], those mothers who do practice KMC may experience a reduction in postpartum depression symptoms [9,47]. They may also experience an increased sense of competence [43]. Evidence from HIC also suggests that KMC has a beneficial impact on overall family dynamics. For example, one study from Israel found family cohesiveness was higher among KMC families as compared to controls [48]. Similarly, qualitative findings from Sweden indicate that KMC "strengthened the mother-father-child unit" [49]. Although further research may be needed to replicate these findings in low- and middle-income countries, it is clear that KMC can be a beneficial intervention not only for the infant, but also for the mother and the family.

### Practicing KMC is often difficult

"Pain / fatigue" emerged as one of the top five barriers to KMC practice when considering all publications and only publications from LMIC. This set of barriers included finding the baby too difficult or heavy to hold [12], discomfort on the chest or back [46], and exhaustion [50], among others. Further, one should note that we identified other barriers that, taken together with the "Pain / fatigue" barrier, indicate that mothers may struggle with the practice. These barriers include "Positioning issues," including difficulty sleeping with the infant on the chest [40], "Breastmilk expression and other breastfeeding-related issues,"[8] discomfort related to temperature [50], and "Issues with clothing / infants' medical devices"[30,51]. Of course, mothers' medical issues also pose a major barrier to practice. These medical issues included pain from episiotomy repair [52], recovery from caesarean section[46], postpartum depression[46], and general maternal illness [12,53].

These barriers suggest that practicing continuous KMC is likely very challenging for mothers, especially those who have low motivation and medical issues.

### Support for mothers can make KMC practice easier

In addition to being physically taxing for mothers, KMC also limits the mother's ability to take care of other tasks and obligations. "Lack of help with KMC practice and other obligations" was ranked among the top five barriers to KMC practice across all publications and when looking only at LMIC. Obligations related to mothers' daily routine came up in publications from countries such as Zimbabwe [40], Uganda [54], Ghana [36], and Sweden [30].

Conversely, "Support from family, friends, and other mothers" emerged as the third-highest-ranked enabler to practice across publications and the top enabler of practice in LMIC. This support took many different forms. Family members would often take turns holding the infant in KMC to give the mother a break from the practice [7,10,55]. They would also take care of other tasks that the mother otherwise would have had to deal with, including childcare and housekeeping [56,57]. Qualitative evidence also indicates that emotional support provides an important, and sometimes crucial, enabler to practice. For example, in Malawi, when looking to overcome issues of fear or embarrassment for the mothers, implementers found, "the most effective way to ensure KMC continues at home is to involve the grandma during the admission" [58]. Similarly critical roles of family members providing emotional support were documented in Ghana [36] and South Africa [39].

Several studies also documented the role that other mothers could play in training or supporting mothers in KMC practice. For example, in a study investigating a community-based application of KMC in Bangladesh, one third of mothers who had been trained on community-initiated KMC reported teaching the practice to others [11]. There is quantitative evidence from Ghana that this phenomenon has an impact on practice; infants in a region where some women had been trained on STS but whose mothers had not been taught STS were more likely to receive STS than infants born in regions where no mothers had been taught STS (RR_Any [STS care]_: 1.28; 95% CI: 0.92–1.79; RR_> 2 h [STS care]_: 1.64; 95% CI: 0.80–3.39), thereby suggesting that mothers discussed their STS practice with each other [37]. Qualitative findings also indicate that KMC mothers support other mothers starting the practice on the ward. In South Africa, for example, KMC mothers supported each other on the ward in various ways: "they reminded each other about the importance of KMC for their babies; discussed how to comfort their babies, and how to kangaroo the infants properly, as demonstrated; and exchanged ideas on how to minimise discomfort" [22]. Similar experiences were found in Mozambique [59] and Mexico, Indonesia, and Ethiopia.

Interestingly, "Support from staff or community health workers" was the fourth-highest-ranked enabler for practice across publications but fell to seventh when looking only at publications from LMIC. Although further research is needed, this finding, combined with the finding that support from family, friends, and other mothers is a top enabler to practice, indicates that the community may play a critical role in promoting KMC practice in low-resource settings. Going forward, it will be important for researchers and implementers to understand how the community can complement a facility-based approach to scale-up with community engagement activities, drive demand for the practice, and ensure infants receive quality KMC care.

### Physical environment and resourcing factors can be barriers to practice, but these are under-studied in the community setting

"Issues with facility environment / resources" emerged as the top barrier to practice for mothers, and this factor includes an array of different issues. These issues included crowdedness and noisiness [22,50,60], lack of privacy [61,62], lack of food and supplies [40,54], and uncomfortable beds [13,22]. It is important to remember that, due to the nature of KMC guidelines, facility-related issues may be over-represented in these findings. Data regarding nurses' barriers to adoption also suggests that resource-related factors, such as workload, play an important role in the implementation of KMC.

It is also important to note that there is a paucity of information available on physical and resourcing barriers to practice for mothers practicing KMC in the community. Of the 103 articles included in this review, only 16 focused on community-initiated KMC or had a substantial focus on community-based practice and perspectives. Thus, although a lack of resources in the community, such as comfortable beds and readily available food, may be an equally common barrier, the data on this topic is currently limited by the focus of existing literature. Of course, institution-initiated KMC is more commonly accepted as an evidence-based practice [3], which may account for some of the lack of research on practice outside the facility. However, because facility and community practice of KMC actually represent a continuum, with infants moving back and forth between the two, there is still opportunity to study community barriers to practice, even within a facility-initiated KMC program [24].

## Directions for Future Research and Practice

This systematic review prioritizes the main factors that influence KMC practice, and, in doing so, highlights some key areas that implementers and implementation researchers may need to focus on when promoting KMC. Given that local circumstances, including cultural attitudes and support for the mother, have an impact on KMC practice, it is critical to understand the context-specific factors that might impact a KMC program. Qualitative and ethnographic research, including interviews with mothers who have practiced KMC and healthcare providers, as well focus groups with community members, can achieve this goal. Implementers should also study the effectiveness of various user-centric designs for promoting KMC, including different mechanisms to ensure the mother has support for practice.

In addition, this review points out the difficulty that mothers have practicing continuous KMC (at least 20 hours of STS / day). Accordingly, more research and analysis is needed to understand the dose-response effect of KMC. If mothers could practice for shorter periods of time without reducing the mortality impact of the practice, KMC might be more feasible and easier to scale. Researchers should re-examine existing data on the number of hours of STS that infants received and the associated mortality impact, as well as track actual STS hours in forthcoming continuous KMC programs in order to compare infants who received at least 20 hours of STS with those who received fewer (ie, infants whose mothers deviated from the protocol).

## Limitations of this Study

This review is limited by definitional challenges related to the practice and implementation of KMC. Since WHO guidelines currently do not recommend community-initiated KMC, there is likely significant bias in the literature toward institution-related barriers to KMC practice [2]. Therefore, it is likely that more research will focus on issues related to providing KMC in the facility than on issues related to the community, such as cultural perceptions of KMC. However, because mothers and newborns require a continuum of care that extends into both the facility and community, there are likely important barriers to the practice of KMC that relate to community beliefs about newborn care which may be underrepresented in this review.

There also exists some inconsistency in the definition of KMC practice. Even studies included in the Cochrane Review's meta-analysis of KMC, which used rigorous publication inclusion criteria and which helped establish KMC as an evidence-based practice for reducing preterm mortality and morbidity, had widely varying applications of KMC [3]. For example, Worku et al. did not require infants to be stabilized before beginning KMC, even though most other studies included in the meta-analysis did [63]. Similarly, the studies included in this meta-analysis had a wide range in the number of hours of STS care actually practiced by mothers and guardians: while some studies reported continuous contact for approximately 20 hours / day [64], others reported an average of only 1–2 hours of STS care / day [65,66]. Unfortunately, dose-response data for KMC is not available. Given that variations in the application of KMC exist and do not always follow WHO guidelines, our review necessarily includes publications that reflect this variation. By incorporating findings from the broadest range of publications which report barriers to KMC practice, including those publications which only sought to implement STS care (the hallmark component of KMC) and not its other components, we believe we have captured the full range of barriers that one could face when implementing a KMC program.

In addition, the majority of papers identified focuses on mothers and excludes fathers' and other family members' perspectives, and they focus on nurses and exclude physicians' perspectives. Although this likely reflects the reality of the situation that mothers practice KMC more often than fathers and nurses train parents on KMC more often than physicians, future research may need to focus on barriers to practice for fathers and physicians.

There is also a risk that the barriers identified across studies are not the most important barriers to practice, but rather the most easily observable barriers. As mentioned, this review is designed to synthesize the literature on barriers to practice in order to serve as a starting point for future research, rather than to determine which barriers are most critical to overcome in order to ensure the maximum number of hours of STS contact. Because this study included qualitative and observational information from many sources, including publications which did not explicitly set out to address the topic of barriers to KMC practice, it would be impossible to determine which of these barriers are most important (ie, in order to increase the number of hours that a mother can practice STS).

Finally, this review is limited by the fact that only studies published in English were included; in particular, there may be data from non-English-speaking LMIC that have relevant information on barriers / enablers to KMC practice which are not included in this review.

## Conclusion

As KMC gains momentum with the rollout of various other Reproductive, Maternal, Newborn, & Child Health and Nutrition programs, including ENAP, it is critical to understand the barriers to practice for the end-users, often the mother, of this life-saving practice, which has many additional benefits for infants and mothers. This systematic review sought to synthesize the most frequently cited barriers to practice for mothers, fathers, CHW's, nurses, physicians, and program managers, as well as the most commonly cited enablers to practice for mothers. The findings from this review can be used to guide future programmatic research efforts aiming to understand how to effectively implement KMC at scale, as well as the design or update of implementation efforts across geographies.

## Supporting Information

S1 Appendix PRISMA checklist. (PDF)

S2 Appendix Detailed methodology for indexed ranking of barriers / enablers. (DOCX)

S3 Appendix Full list of publications included in analysis for systematic review. (DOCX)

S1 Dataset Complete dataset used for analysis in systematic review. (XLSX)
