# Supplementary material for: Barriers and Enablers of Kangaroo Mother Care Practice: A Systematic Review
Source: PLoS One. 2015 May 20;10(5):e0125643. doi: 10.1371/journal.pone.0125643 (PMC4439040; doi:10.1371/journal.pone.0125643)
Supplement: S2 Appendix — (DOCX) [file pone.0125643.s002.docx]

**Appendix S2 – Detailed methodology for indexed ranking of barriers / enablers**

**Background**

The goal of this systematic review was twofold. First, we set out to identify all the barriers and enablers to KMC discussed in the relevant literature. In addition, we aimed to synthesize these barriers in a systematic way in order to identify those barriers that were most commonly identified and therefore most likely to be relevant for implementers designing KMC and related programs. Accordingly, we decided to include in our review any publication that described barriers to KMC or skin-to-skin [STS] practice. By allowing for inclusion of many publications, including those that did not study barriers using a quantitative approach, we were able to ensure that our data captured barriers that were noted only as observational findings.

On the other hand, we recognize that the relevance of the findings on barriers to KMC is likely much higher from those publications that systematically set out to document these factors than those which identified them through exploratory or indirect means. Accordingly, we developed an indexed ranking methodology which gave greater weight to barriers identified in more rigorous research.

**Indexed ranking methodology**

Our indexed ranking methodology involved classifying each publication as one of four types, based on the rigor of the research with regards to identification of barriers / enablers to practice. These four categories, in descending order of rigor, are: Prioritized, Systematic, Exploratory, and Indirect. A definition of each of these barriers can be found in the table below.

Our data collection methodology counted the frequency of each category of barriers / enablers to practice. In order to ensure that those barriers / enablers cited in research that had a more systematic approach to identifying them were weighted more heavily than those from less systematic research, we assigned a weighting value to each publication type: Prioritized – 4, Systematic – 3, Exploratory – 2, Indirect – 1. In other words, a barrier identified in a Prioritized study was counted four times, whereas a barrier identified in an Indirect study was only counted once.

Because of the nature of Prioritized studies, we were also able to exclude barriers that were of low importance. For any barrier identified in a Prioritized study, the barrier had to impact greater than 20% of the population in order to be considered a relevant data point.

| **Publication category** | **Study description** | **Weighting value** |
| --- | --- | --- |
| Indirect | Studies that do not set out to identify barriers / enablers to KMC practice but which mention these factors in their findings | 1 |
| Exploratory | Studies that set out to identify barriers / enablers to practice or experiences with KMC but do not pre-specify the factors under consideration | 2 |
| Systematic | Studies that set out to identify barriers / enablers to practice and that pre-specify the specific barriers under consideration in a systematic way but do not prioritize these barriers to practice as part of their findings | 3 |
| Prioritized | Studies that set out to identify barriers / enablers to practice, pre-specify the specific barriers under consideration in a systematic way, and prioritize these barriers to practice as part of their findings | 4 |

**Data collection template**

Our data collection template included a row for every publication. For each publication, whenever evidence of a barrier / enabler was identified, this barrier / enabler was categorized into one of the categories for barriers / enablers that had been identified during the piloting of the data collection template. The barrier / enabler was coded with a “1”. The data collection template was maintained in Microsoft Excel. This software was also used for the tabulation and weighting of all barriers.

A representative screenshot of the data collection template used can be found below.


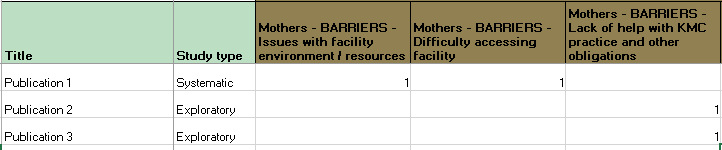


**Example of indexed ranking methodology**

It is instructive to provide an example of how the indexed ranking methodology works with data from actual publications. In the screenshot above, data from three publications for three barriers is shown. Publication 1 had evidence of two barriers – "Issues with facility environment / resources" and "Difficulty accessing facility". Publications 2 and 3 each had evidence of one barrier – "Lack of help with KMC and other obligations". Evidence of this barrier was coded by including the exact text in the appropriate cell in the data collection template.

Publication 1 was categorized as a "Systematic" publication, meaning that each barrier identified from this publication received a weight of 3. Publications 2 and 3 were both categorized as "Exploratory" publications, meaning that each barrier identified from these publications received a weight of 2. The final indexed ranking of these three barriers from these three publications would be as follows:

| **Barrier** | **Indexed ranking** |
| --- | --- |
| Issues with facility environment / resources | 3 |
| Difficulty accessing facility | 3 |
| Lack of help with KMC practice and other obligations | 4 |

**Definition of barrier / enabler groupings**

Below are definitions for the three groupings for the barriers / enablers identified in this study.

| Grouping | Definition |
| --- | --- |
| Resourcing | Barriers: any barrier which would need to be overcome by changing the quality or quantity of resources dedicated to supporting KMC practice  Enablers: any enabler related to the provision of resources or support that a parent receives when providing KMC |
| Sociocultural | Any barrier / enabler related to the unique aspects of the culture in which the parent was living or practicing KMC; in contrast to experiential barriers / enablers, these factors were more general to society, and less specific to the individual |
| Experiential | Any barrier / enabler directly related to the experience of practicing KMC; in contrast to sociocultural barriers / enablers, factors listed in this category were specific to the individual, rather than broader society |
