# Supplementary material for: Barriers and Enablers of Kangaroo Mother Care Practice: A Systematic Review
Source: PLoS One. 2015 May 20;10(5):e0125643. doi: 10.1371/journal.pone.0125643 (PMC4439040; doi:10.1371/journal.pone.0125643)
Supplement: S3 Appendix — (DOCX) [file pone.0125643.s003.docx]

**Appendix S3 - Full list of publications included in review**

| **Title** | **Author** | **Journal** | **Year** | **Categorization*** |
| --- | --- | --- | --- | --- |
| The breastfeeding support and promotion in Baby-Friendly Maternity Hospitals and Not-as-Yet Baby-Friendly Hospitals in Russia | Abolyan, L. V. | Breastfeed Med | 2006 | 3 |
| Evaluation of mothers' knowledge, attitudes, and practice towards the ten steps to successful breastfeeding in Egypt | Abul-Fadl, A. M.; Shawky, M.; El-Taweel, A.; Cadwell, K.; Turner-Maffei, C. | Breastfeed Med | 2012 | 3 |
| Community Kangaroo Mother Care: implementation and potential for neonatal survival and health in very low-income settings | Ahmed, S.; Mitra, S. N.; Chowdhury, A. M.; Camacho, L. L.; Winikoff, B.; Sloan, N. L. | J Perinatol | 2011 | 1 |
| Mother-newborn contact in a randomized trial of kangaroo (skin-to-skin) care | Anderson, G. C.; Chiu, S. H.; Dombrowski, M. A.; Swinth, J. Y.; Albert, J. M.; Wada, N. | J Obstet Gynecol Neonatal Nurs | 2003 | 2 |
| Kangaroo mother method: Mothers' experiences and contributions to nursing | Arivabene, J. C.; Tyrrell, M. A. R. | Revista Latino-Americana de Enfermagem | 2010 | 2 |
| Making Kangaroo Care the Norm: Implementation of a New Model of Care | Barabach, L.; Sedlock, J.; Salmon, K. | Jognn-Journal of Obstetric Gynecologic and Neonatal Nursing | 2012 | 1 |
| Introducing home based skin-to-skin care for low birth weight newborns: a pilot approach to education and counseling in Ghana | Bazzano, A.; Hill, Z.; Tawiah-Agyemang, C.; Manu, A.; ten Asbroek, G.; Kirkwood, B. | Global Health Promotion | 2012 | 2 |
| Translating research findings into practice--the implementation of kangaroo mother care in Ghana | Bergh, A. M.; Manu, R.; Davy, K.; van Rooyen, E.; Asare, G. Q.; Williams, J. K.; Dedzo, M.; Twumasi, A.; Nang-Beifubah, A. | Implement Sci | 2012 | 2 |
| Progress with the implementation of kangaroo mother care in four regions in Ghana | Bergh, A. M.; Manu, R.; Davy, K.; Van Rooyen, E.; Quansah Asare, G.; Awoonor-Williams, J.; Dedzo, M.; Twumasi, A.; Nang-Beifubah, A. | Ghana Med J | 2013 | 3 |
| Scaling up kangaroo mother care in South Africa: 'on-site' versus 'off-site' educational facilitation | Bergh, A. M.; van Rooyen, E.; Pattinson, R. C. | Hum Resour Health | 2008 | 3 |
| The 'kangaroo-method' for treating low birth weight babies in a developing country | Bergman, N. J.; Jurisoo, L. A. | Trop Doct | 1994 | 1 |
| The relation between early mother-infant skin-to-skin contact and later maternal sensitivity in South African mothers of low birth weight infants | Bigelow, A. E.; Littlejohn, M.; Bergman, N.; McDonald, C. | Infant Mental Health Journal | 2010 | 3 |
| Effect of Mother/Infant Skin-to-Skin Contact on Postpartum Depressive Symptoms and Maternal Physiological Stress | Bigelow, A.; Power, M.; MacLellan-Peters, J.; Alex, M.; McDonald, C. | Jognn-Journal of Obstetric Gynecologic and Neonatal Nursing | 2012 | 3 |
| Safety, effectiveness and barriers to follow-up using an 'early discharge' Kangaroo Care policy in a resource poor setting | Blencowe, H.; Kerac, M.; Molyneux, E. | J Trop Pediatr | 2009 | 4 |
| Setting up Kangaroo Mother Care at Queen Elizabeth Central Hospital, Blantyre - A practical approach | Blencowe, Hannah and Molyneux, Elizabeth | Malawi Med Journal | 2005 | 2 |
| Provision of Kangaroo Mother Care: supportive factors and barriers perceived by parents | Blomqvist, Y. T.; Frolund, L.; Rubertsson, C.; Nyqvist, K. H. | Scand J Caring Sci | 2013 | 3 |
| Swedish mothers' experience of continuous Kangaroo Mother Care | Blomqvist, Y. T.; Nyqvist, K. H. | J Clin Nurs | 2011 | 2 |
| Kangaroo Mother Care helps fathers of preterm infants gain confidence in the paternal role | Blomqvist, Y. T.; Rubertsson, C.; Kylberg, E.; Joreskog, K.; Nyqvist, K. H. | J Adv Nurs | 2012 | 2 |
| Effect of early skin-to-skin mother--infant contact during the first 3 hours following birth on exclusive breastfeeding during the maternity hospital stay | Bramson, L.; Lee, J. W.; Moore, E.; Montgomery, S.; Neish, C.; Bahjri, K.; Melcher, C. L. | J Hum Lact | 2010 | 1 |
| A Realistic Evaluation of Two Training Programs on Implementing Skin-to-Skin as a Standard of Care | Brimdyr, K.; Widstrom, A. M.; Cadwell, K.; Svensson, K.; Turner-Maffei, C. | J Perinat Educ | 2012 | 3 |
| Perceptions among post-delivery mothers of skin-to-skin contact and newborn baby care in a periurban hospital in Uganda | Byaruhanga, R. N.; Bergstrom, A.; Tibemanya, J.; Nakitto, C.; Okong, P. | Midwifery | 2008 | 2 |
| Skin-to-skin contact of fullterm infants: an explorative study of promoting and hindering factors in two Nordic childbirth settings | Calais, E.; Dalbye, R.; Nyqvist, Kh; Berg, M. | Acta Paediatr | 2010 | 4 |
| Kangaroo mother care for low birthweight infants: a randomized controlled trial in different settings | Cattaneo, A.; Davanzo, R.; Worku, B.; Surjono, A.; Echeverria, M.; Bedri, A.; Haksari, E.; Osorno, L.; Gudetta, B.; Setyowireni, D.; Quintero, S.; Tamburlini, G. | Acta Paediatr | 1998 | 3 |
| Resistance to implementing Kangaroo Mother Care in developing countries, and proposed solutions | Charpak, N.; Ruiz-Pelaez, J. G. | Acta Paediatr | 2006 | 3 |
| The attitudes and practices of neonatal nurses in the use of kangaroo care | Chia, P.; Sellick, K.; Gan, S. | Aust J Adv Nurs | 2006 | 4 |
| Assessing midwives' breastfeeding knowledge: properties of the Newborn Feeding Ability questionnaire and Breastfeeding Initiation Practices scale | Creedy, D. K.; Cantrill, R. M.; Cooke, M. | Int Breastfeed J | 2008 | 3 |
| Mothers' experiences of skin-to-skin care of healthy full-term newborns - A phenomenology study | Dalbye, R.; Calais, E.; Berg, M. | Sexual and Reproductive Healthcare | 2011 | 2 |
| Introduction of community-based skin-to-skin care in rural Uttar Pradesh, India | Darmstadt, G. L.; Kumar, V.; Yadav, R.; Singh, V.; Singh, P.; Mohanty, S.; Baqui, A. H.; Bharti, N.; Gupta, S.; Misra, R. P.; Awasthi, S.; Singh, J. V.; Santosham, M. | J Perinatol | 2006 | 2 |
| Feasibility of using kangaroo (skin-to-skin) care with colicky infants | Ellett, M. L.; Bleah, D. A.; Parris, S. | Gastroenterol Nurs | 2004 | 1 |
| Kangaroo care: national survey of practice, knowledge, barriers, and perceptions | Engler, A. J.; Ludington-Hoe, S. M.; Cusson, R. M.; Adams, R.; Bahnsen, M.; Brumbaugh, E.; Coates, P.; Grieb, J.; McHargue, L.; Ryan, D. L.; Settle, M.; Williams, D. | MCN Am J Matern Child Nurs | 2002 | 4 |
| The father at the bedside: patterns of involvement in the NICU | Feeley, N.; Sherrard, K.; Waitzer, E.; Boisvert, L. | J Perinat Neonatal Nurs | 2013 | 2 |
| Testing a family intervention hypothesis: the contribution of mother-infant skin-to-skin contact (kangaroo care) to family interaction, proximity, and touch | Feldman, R.; Weller, A.; Sirota, L.; Eidelman, A. I. | J Fam Psychol | 2003 | 3 |
| Influence of NICU co-care facilities and skin-to-skin contact on maternal stress in mothers of preterm infants | Flacking, R.; Thomson, G.; Ekenberg, L.; Lowegren, L.; Wallin, L. | Sex Reprod Healthc | 2013 | 3 |
| Neonatal nurses' knowledge and beliefs regarding kangaroo care with preterm infants in an Irish neonatal unit | Flynn, A.; Leahy-Warren, P. | Journal of Neonatal Nursing | 2010 | 3 |
| Infant holding policies and practices in neonatal units | Franck, L. S.; Bernal, H.; Gale, G. | Neonatal Netw | 2002 | 4 |
| KMC facilitates mother baby attachment in low birth weight infants | Gathwala, G.; Singh, B.; Balhara, B. | Indian J Pediatr | 2008 | 3 |
| Effect of Kangaroo Mother Care on physical growth, breastfeeding and its acceptability | Gathwala, G.; Singh, B.; Singh, J. | Trop Doct | 2010 | 3 |
| Avaliação da implantação do cuidado humanizado aos recém-nascidos com baixo peso: método canguru; Evaluation of implementation of humanized care to low weight newborns: the Kangaroo Method | Gontijo, Tarcísio L.; Meireles, Adriana L.; Malta, Deborah C.; Proietti, Fernando A.; Xavier, César C. | J Pediatr (Rio J) | 2010 | 3 |
| Factors associated with maternal visitation and participation in skin-to-skin care in an all referral level IIIc NICU | Gonya, J.; Nelin, L. D. | Acta Paediatr | 2013 | 3 |
| Kangaroo care in pre-term or low birth weight babies in a postnatal ward | Gregson, S.; Blacker, J. | British Journal of Midwifery | 2011 | 3 |
| Kangaroo Mother Care (KMC) in LBW infants - A western Rajasthan experience | Gupta, M.; Jora, R.; Bhatia, R. | Indian Journal of Pediatrics | 2007 | 2 |
| The role of fathers in the postpartum period: experiences with skin to skin method | Gutierrez, J. J. B.; Perez, M. D. A.; Aguilera, M. V. M.; Moreno, S. G. | Acta Paulista De Enfermagem | 2012 | 2 |
| Implementing skin-to-skin contact at birth using the Iowa model: applying evidence to practice | Haxton, D.; Doering, J.; Gingras, L.; Kelly, L. | Nurs Womens Health | 2012 | 2 |
| Factors affecting parents' presence with their extremely preterm infants in a neonatal intensive care room | Heinemann, A. B.; Hellstrom-Westas, L.; Hedberg Nyqvist, K. | Acta Paediatr | 2013 | 1 |
| Factors that influence neonatal nursing perceptions of family-centered care and developmental care practices | Hendricks-Munoz, K. D.; Louie, M.; Li, Y.; Chhun, N.; Prendergast, C. C.; Ankola, P. | Am J Perinatol | 2010 | 3 |
| Keeping newborns warm: beliefs, practices and potential for behaviour change in rural Ghana | Hill, Z.; Tawiah-Agyemang, C.; Manu, A.; Okyere, E.; Kirkwood, B. R. | Trop Med Int Health | 2010 | 2 |
| Factors influencing implementation of kangaroo holding in a Special Care Nursery | Johnson, A. N. | MCN Am J Matern Child Nurs | 2007 | 4 |
| The maternal experience of kangaroo holding | Johnson, A. N. | J Obstet Gynecol Neonatal Nurs | 2007 | 2 |
| Feasibility of kangaroo mother care in Mumbai | Kadam, S.; Binoy, S.; Kanbur, W.; Mondkar, J. A.; Fernandez, A. | Indian J Pediatr | 2005 | 3 |
| Kangaroo care for well low birth weight infants at Harare central hospital maternity unit - Zimbabwe | Kambarami, R. A.; Chidede, O.; Kowo, D. T. | Central African Journal of Medicine | 1999 | 1 |
| Caregivers' perceptions and experiences of 'kangaroo care' in a developing country | Kambarami, R. A.; Mutambirwa, J.; Maramba, P. P. | Trop Doct | 2002 | 2 |
| Women's authority during childbirth and Safe Motherhood in Yemen | Kempe, A.; Noor-Aldin Alwazer, F. A.; Theorell, T. | Sex Reprod Healthc | 2010 | 3 |
| Effect of the Newhints home-visits intervention on neonatal mortality rate and care practices in Ghana: a cluster randomised controlled trial | Kirkwood, B. R.; Manu, A.; ten Asbroek, A. H.; Soremekun, S.; Weobong, B.; Gyan, T.; Danso, S.; Amenga-Etego, S.; Tawiah-Agyemang, C.; Owusu-Agyei, S.; Hill, Z. | Lancet | 2013 | 3 |
| Mother-infant interaction is influenced by the amount of holding in preterm infants | Korja, R.; Maunu, J.; Kirjavainen, J.; Savonlahti, E.; Haataja, L.; Lapinleimu, H.; Manninen, H.; Piha, J.; Lehtonen, L.; Pipari Study Grp | Early Human Development | 2008 | 3 |
| Effect of community-based behaviour change management on neonatal mortality in Shivgarh, Uttar Pradesh, India: a cluster-randomised controlled trial | Kumar, V.; Mohanty, S.; Kumar, A.; Misra, R. P.; Santosham, M.; Awasthi, S.; Baqui, A. H.; Singh, P.; Singh, V.; Ahuja, R. C.; Singh, J. V.; Malik, G. K.; Ahmed, S.; Black, R. E.; Bhandari, M.; Darmstadt, G. L. | Lancet | 2008 | 3 |
| Balancing preterm infants' developmental needs with parents' readiness for skin-to-skin care: A phenomenological study | Kymre, I. G.; Bondas, T. | International Journal of Qualitative Studies on Health and Well-being | 2013 | 2 |
| Clinician perspectives on barriers to and opportunities for skin-to-skin contact for premature infants in neonatal intensive care units | Lee, H. C.; Martin-Anderson, S.; Dudley, R. A. | Breastfeeding Medicine | 2012 | 2 |
| Kangaroo care in a neonatal context: parents' experiences of information and communication of nurse-parents | Lemmen, D.; Fristedt, P.; Lundqvist, A. | Open Nurs J | 2013 | 2 |
| Parents' Lived Experience of Providing Kangaroo Care to their Preterm Infants | Leonard, A.; Mayers, P. | Health SA Gesondheid | 2008 | 2 |
| The course of childbirth and the contact STS of mother with child and the symptoms of postpartum depression | Lessing-Pernak, J. | Ginekologia i Poloznictwo | 2011 | 3 |
| Feasibility, acceptability and cost of kangaroo mother care in Recife, Brazil | Lima, G.; Quintero-Romero, S.; Cattaneo, A. | Ann Trop Paediatr | 2000 | 3 |
| Kangaroo mother care with limited resources | Lincetto, O.; Nazir, A. I.; Cattaneo, A. | J Trop Pediatr | 2000 | 1 |
| Breastfeeding Support in Neonatal Intensive Care: A National Survey | Maastrup, R.; Bojesen, S. N.; Kronborg, H.; Hallstrom, I. | Journal of Human Lactation | 2012 | 3 |
| Implementing a Birth Kangaroo Care Policy in Labor and Delivery: Bringing Evidence-Based Practice to the Bedside | Maloof-Bury, P. A.; Russell, E. | Jognn-Journal of Obstetric Gynecologic and Neonatal Nursing | 2013 | 1 |
| Influence of an education program of pregnant women on delivery | Martinez-Galiano, J. M.; Delgado-Rodriguez, M. | J Matern Fetal Neonatal Med | 2013 | 3 |
| Kangaroo care in Port Moresby, Papua New Guinea | McMaster, P.; Haina, T.; Vince, J. D. | Trop Doct | 2000 | 2 |
| Effect of kangaroo mother care on premature infants' physiological, behavioral and psychosocial outcomes in Ain Shams maternity and Gynecological Hospital, Cairo, Egypt | Mohammed El-Nagger, N. S.; Abed El-Azim, H.; Mahmoud Zaki Hassan, S. | Life Science Journal | 2013 | 3 |
| Maternal kangaroo (skin-to-skin) care in the NICU beginning 4 hours postbirth | Moran, M.; Radzyminski, S. G.; Higgins, K. R.; Dowling, D. A.; Miller, M. J.; Anderson, G. C. | MCN Am J Matern Child Nurs | 1999 | 1 |
| Knowledge and Awareness about Benefits of Kangaroo Mother Care | Muddu, G. K.; Boju, S. L.; Chodavarapu, R. | Indian J Pediatr | 2013 | 3 |
| Observations on kangaroo baby care | Mukasa, G. K. | Mothers Child | 1992 | 1 |
| Kangaroo care: is it for everyone? | Neu, M. | Neonatal Netw | 2004 | 2 |
| Parents' perception of skin-to-skin care with their preterm infants requiring assisted ventilation | Neu, M. | J Obstet Gynecol Neonatal Nurs | 1999 | 2 |
| Perception and practice of Kangaroo Mother Care after discharge from hospital in Kumasi, Ghana: a longitudinal study | Nguah, S. B.; Wobil, P. N.; Obeng, R.; Yakubu, A.; Kerber, K. J.; Lawn, J. E.; Plange-Rhule, G. | BMC Pregnancy Childbirth | 2011 | 3 |
| Kangaroo Mother Care: Effect and perception of mothers and health personnel | Nirmala, P.; Rekha, S.; Washington, M. | Journal of Neonatal Nursing | 2006 | 3 |
| Application of the baby friendly hospital initiative to neonatal care: suggestions by Swedish mothers of very preterm infants | Nyqvist, K. H.; Kylberg, E. | J Hum Lact | 2008 | 3 |
| Mothers' advice about facilitating breastfeeding in a neonatal intensive care unit | Nyqvist, K. H.; Sjoden, P. O.; Ewald, U. | J Hum Lact | 1994 | 4 |
| Skin-to-skin care in neonatal intensive care units in the Nordic countries: a survey of attitudes and practices | Olsson, E.; Andersen, R. D.; Axelin, A.; Jonsdottir, R. B.; Maastrup, R.; Eriksson, M. | Acta Paediatr | 2012 | 4 |
| Parental involvement and kangaroo care in European neonatal intensive care units: a policy survey in eight countries | Pallas-Alonso, C. R.; Losacco, V.; Maraschini, A.; Greisen, G.; Pierrat, V.; Warren, I.; Haumont, D.; Westrup, B.; Smit, B. J.; Sizun, J.; Cuttini, M. | Pediatr Crit Care Med | 2012 | 3 |
| Experience with Kangaroo mother care in a neonatal intensive care unit (NICU) in Chandigarh, India | Parmar, V. R.; Kumar, A.; Kaur, R.; Parmar, S.; Kaur, D.; Basu, S.; Jain, S.; Narula, S. | Indian J Pediatr | 2009 | 3 |
| Implementation of kangaroo mother care: a randomized trial of two outreach strategies | Pattinson, R. C.; Arsalo, I.; Bergh, A. M.; Malan, A. F.; Patrick, M.; Phillips, N. | Acta Paediatr | 2005 | 3 |
| Adaptation of kangaroo mother care for community-based application | Quasem, I.; Sloan, N. L.; Chowdhury, A.; Ahmed, S.; Winikoff, B.; Chowdhury, A. M. | J Perinatol | 2003 | 2 |
| Getting to know you: mothers' experiences of kangaroo care | Roller, C. G. | J Obstet Gynecol Neonatal Nurs | 2005 | 2 |
| Kangaroo Care for a restless infant with gastric reflux. One nurse midwife's personal experience | Roller, C. G. | MCN Am J Matern Child Nurs | 1999 | 1 |
| Birth kangaroo (skin-to-skin) care and breastfeeding. An eclamptic woman's story | Roller, C. G.; Meyer, K.; Anderson, G. C. | MCN Am J Matern Child Nurs | 1999 | 1 |
| Neonatal care in the home in northern rural Honduras: a qualitative study of the role of traditional birth attendants | Sacks, E.; Bailey, J. M.; Robles, C.; Low, L. K. | J Perinat Neonatal Nurs | 2013 | 2 |
| Utilization of postnatal care for newborns and its association with neonatal mortality in India: an analytical appraisal | Singh, A.; Yadav, A.; Singh, A. | BMC Pregnancy Childbirth | 2012 | 2 |
| Effectiveness of a structured teaching programme on Kangaroo care among the mothers of preterm babies | Sivapriya, S.; Subash, J.; Kamala, S. | Perinatology | 2007 | 3 |
| Community-based kangaroo mother care to prevent neonatal and infant mortality: a randomized, controlled cluster trial | Sloan, N. L.; Ahmed, S.; Mitra, S. N.; Choudhury, N.; Chowdhury, M.; Rob, U.; Winikoff, B. | Pediatrics | 2008 | 3 |
| Knowledge and attitudes of nursing staff and mothers towards kangaroo mother care in the eastern sub-district of Cape Town | Solomons, N.; Rosant, C. | South African Journal of Clinical Nutrition | 2012 | 3 |
| Applying the plan-do-study-act model to increase the use of kangaroo care | Stikes, R.; Barbier, D. | J Nurs Manag | 2013 | 4 |
| Kangaroo mother care for low birth weight infants: a randomized controlled trial | Suman, R. P.; Udani, R.; Nanavati, R. | Indian Pediatr | 2008 | 3 |
| Towards better care for preterm infants in bamako, Mali | Sylla, M.; Kassogue, D.; Traore, I.; Diall, H.; Charpak, N.; Dicko-Traore, F.; N'Diaye, M.; Doumbia, D.; Kamissoko, F.; Sidibe, T.; Keita, M. M. | Current Women's Health Reviews | 2011 | 3 |
| Kangaroo mother care and the bonding hypothesis | Tessier, R.; Cristo, M.; Velez, S.; Giron, M.; de Calume, Z. F.; Ruiz-Palaez, J. G.; Charpak, Y.; Charpak, N. | Pediatrics | 1998 | 3 |
| Reducing neonatal infections in south and south central Vietnam: the views of healthcare providers | Trevisanuto, D.; Arnolda, G.; Chien, T. D.; Xuan, N. M.; Thu le, T. A.; Kumara, D.; Lincetto, O.; Moccia, L. | BMC Pediatr | 2013 | 1 |
| Community neonatal practices and its association with skilled birth attendance in rural Haryana, India | Upadhyay, R. P.; Rai, S. K.; Anand, K. | Acta Paediatr | 2012 | 3 |
| Nurses' viewpoint about the impact of Kangaroo Mother Care on the mother-infant attachment | Valizadeh, L.; Ajoodaniyan, N.; Namnabati, M.; Zamanzadeh, V.; layegh, V. | Journal of Neonatal Nursing | 2013 | 3 |
| Promoting skin-to-skin care for low birthweight babies: findings from the Ghana Newhints cluster-randomised trial | Vesel, L.; Ten Asbroek, A. H.; Manu, A.; Soremekun, S.; Tawiah Agyemang, C.; Okyere, E.; Owusu-Agyei, S.; Hill, Z.; Kirkwood, B. R. | Trop Med Int Health | 2013 | 2 |
| Implementation of kangaroo care: a parent-health care team approach to practice change | Victor, L.; Persoon, J. | Crit Care Nurs Clin North Am | 1994 | 2 |
| I never thought that this baby would survive; I thought that it would die any time': perceptions and care for preterm babies in eastern Uganda | Waiswa, P.; Nyanzi, S.; Namusoko-Kalungi, S.; Peterson, S.; Tomson, G.; Pariyo, G. W. | Trop Med Int Health | 2010 | 2 |
| Staff experiences in implementing guidelines for Kangaroo Mother Care - A qualitative study | Wallin, L.; Rudberg, A.; Gunningberg, L. | International Journal of Nursing Studies | 2005 | 3 |
| Randomized controlled trial of Family Nurture Intervention in the NICU: assessments of length of stay, feasibility and safety | Welch, M. G.; Hofer, M. A.; Stark, R. I.; Andrews, H. F.; Austin, J.; Glickstein, S. B.; Ludwig, R. J.; Myers, M. M. | BMC Pediatr | 2013 | 3 |
| Kangaroo mother care: a randomized controlled trial on effectiveness of early kangaroo mother care for the low birthweight infants in Addis Ababa, Ethiopia | Worku, B.; Kassie, A. | J Trop Pediatr | 2005 | 1 |
| Effectiveness of the UNICEF/WHO 20-hour course in improving health professionals' knowledge, practices, and attitudes to breastfeeding: Before/after study of 5 maternity facilities in croatia | Zakarija-Grkovic, I.; Burmaz, T. | Croatian Medical Journal | 2010 | 3 |

***Note:** Code for study categorization: 1=Indirect; 2=Exploratory; 3=Systematic; 4=Prioritized
